# Supplementary material for: Patterns of Intron Gain and Loss in Fungi
Source: PLoS Biol. 2004 Nov 30;2(12):e422. doi: 10.1371/journal.pbio.0020422 (PMC532390; doi:10.1371/journal.pbio.0020422)
Supplement: Table S1 — Also available at http://genes.mit.edu/NielsenEtAl/. (4.3 MB ZIP). [file pbio.0020422.st001.zip › NielsenEtAl/html/430.html]

AN9090.1.NCU00768.1.MG08741.1.FG05737.1


```
 CLUSTAL W (1.82) Multiple Sequence Alignments - Introns Inserted


Sequence 1: NCU00768.1	491 aa
Sequence 2: MG08741.1	415 aa
Sequence 3: FG05737.1	409 aa
Sequence 4: AN9090.1	393 aa
Alignment Length: 528 aa
Number Identitical Residues: 229 aa
Alignment Score (without introns) 9953


MG08741.1 	---------MSTFPPPTPQGNGHAGDN--GNAMNGSGAQMEGNFQTPPV-----VPQG--
NCU00768.1	---------MADYTAPGAPGTNNAAP-----TMNGN---SEANFQAPAA-----LVQPGT
FG05737.1 	---------MDMNPGFSGVPANNGAPS--GPMAYSNGTPPAPDMGGVAP-----PPGSGP
AN9090.1  	MSFQNFDSFQNQHPAADAAATAPGAPATADTTMTGQADPSTAPFQGPAPGEGAPAVAQQG
          	 : .. .:     .         ..  ::.    ...      :   . ...:.      

MG08741.1 	NDSAKTLW2MGELEPWMDENFIKQIWSTVCGETVNVKVIRDRQSG2--NAGYCFVEFPSS
NCU00768.1	NEASKTLW2MGEMEGWMDENFIKNVFTTVLGETVQVKVIRDRNSG2--NAGYCFIEFATP
FG05737.1 	DSPKTTLW2MGELEPWMDENFIKGVFLSSAHENVNVKVIRDKNSG~--NAGYCFVEFQSP
AN9090.1  	NEGKTTLW2MGELEPWIDENFVRNLWFQMG-EQVNVKMIRDKFSG2RSNAGYCFVDFASP
          	:.  .*** ***:* *:****:: ::     * *:**:***: **  :******::* :.

MG08741.1 	EAATKALGLNGSPVPNSQRVFKLNWASGGGLVDR-R2DERGPEYSIFVGDLGPEVNEFVL
NCU00768.1	EAAQKALNLNGTPVPNSNRAFKLNWASGGGLIDR-R2DDRGPEYSIFVGDLGPEVNEFVL
FG05737.1 	EAATKALNMNGSQVPNSSRSFKLNWASGGGLVDR-R2DDRGPEYSIFVGDLGPEVNEYVL
AN9090.1  	AAAAKALSLNGTPMPNTNRLFKLNWATGGGLADRSR~DDRGPEYSIFVGDLGPEVNEYVL
          	 ** ***.:**: :**:.* ******:**** **:* *:******************:**

MG08741.1 	VSLFQARFPSCKSAKIMTDAMTGQSRGYGFVRFTDESDQQRALVEMQGVYCGNRPMRIST
NCU00768.1	VSLFQSRFPSCKSAKIMTDAMTGQSRGYGFVRFSDEGDQQRALVEMQGVYCGNRPMRIST
FG05737.1 	VSLFQARFPSCKSAKIMTDAMSGQSRGYGFVRFSDENDQQRALVEMQGVYCGNRPMRIST
AN9090.1  	VSLFQSRFPSCKSAKIMTDPISGMSRGYGFVRFSDENDQQRALSEMQGVYCGNRPMRIST
          	*****:*************.::* *********:**.****** ****************

MG08741.1 	ATPKTR2SHQQYGNQGPHGAGPMNMTPP--AQNMQWGMNPYGYQQPQAPPPNTAFNNPMQ
NCU00768.1	ATPKTR2TH-QYGAHAPHAANPMMAPVPAHATNLQWGVPPQ--------PYYSGFN-PMQ
FG05737.1 	ATPKNR~GNHGFGGQGHQHGGPMMPGMP--QQQMWNGGGMQG-------FPYGGFN-PAT
AN9090.1  	ATPKNK~GPGVVPG-GMGMPGPAGMYPP-------MGAPPMG------------FYGAPQ
          	****.:         .    .*     *        *    .            * ..  

MG08741.1 	PMNQFTDPNNTTVFVGGLSGYVTEDELRSFFQGFGDITYVKIPPGKGCGFVQFVHRHAAE
NCU00768.1	PMNQFTDPNNTTVFVGGLSGYVTEDELRSFFQGFGEITYVKIPPGKGCGFVQFVHRHAAE
FG05737.1 	QMNQFTDPNNTTVFVGGLSGYVTEDELRSFFQGFGEITYVKIPPGKGCGFVQFVHRHAAE
AN9090.1  	PMNQFTDPNNTTVFVGGLSGYVTEDELRSFFQGFGEITYVKIPPGKGCGFVQFVQRHAAE
          	 **********************************:******************:*****

MG08741.1 	MAINQMQGYPIGNSRVRLSWGRSQNNSG-VGTPYRPAPPPPHWGGGMPPHGGPAGP-GAP
NCU00768.1	MAINQMQGYPIGNSRVRLSWGRSQNNSG-VGTPYRPAPPPPHYMGPPPPPG-PAGPYGAP
FG05737.1 	MAINQMQGYPIGNSRVRLSWGRSQNNSG-VGTPYRPAPPPPHYMG-MPSHG-GPGNYGPQ
AN9090.1  	MAINQMQGYPIGNSRVRLSWGRSQNNSGPAGSPYRPAPPPP--PMYLPPQH---------
          	****************************..*:*********      *.           

MG08741.1 	FGGPFGGNP~P--------------PPPGGLQVSPQPPI-~-------------------
NCU00768.1	HFGGNGGPP~PALVGPTSSDQYTPAPSPAQLAGSPQPPAT~PVLAYATLYTVTPPPVTSE
FG05737.1 	HFGGPAPGP~Q--------------GPPGPPGPGPNGPPQ0-------------------
AN9090.1  	QYGGFAPMK0------------------------------~-------------------
          	  *  .                                                      

MG08741.1 	------------------------------------------------------
NCU00768.1	ASNPGRRRQPTSRVAGGRVGSNGRVLSGRITKPKTEPSGSNGQVGRFFRRGGMK
FG05737.1 	------------------------------------------------------
AN9090.1  	------------------------------------------------------
          	
```
